# Not all carotenoids can reduce the risk of gastric cancer: a systematic review with meta-analysis

**DOI:** 10.1186/s12876-024-03139-5

**Published:** 2024-01-29

**Authors:** Wei Han, Wei Zhang, Xuan Ren

**Affiliations:** 1 Department of General Surgery, Lanzhou University Second Hospital, Lanzhou, 730030 China; 2 The Second Clinical Medical College of Lanzhou University, Lanzhou, 730000 China; 3 Department of Endocrinology and Metabolism, Lanzhou University Second Hospital, Lanzhou, 730030 China

**Keywords:** Carotenoids, Gastric cancer, Risk, Systematic review, Meta-analysis

## Abstract

**Background:**

Gastric cancer is characterized by high invasiveness, heterogeneity, and late diagnosis, leading to high incidence and mortality rates. It is a significant public health concern globally. Early prevention is crucial in reducing the occurrence of gastric cancer, and dietary prevention, particularly focusing on carotenoids, has been considered a convenient and effective approach. However, the association between carotenoid intake and gastric cancer incidence remains controversial.

**Methods:**

A systematic search was conducted in PubMed, Ovid Embase, Web of Science, and Cochrane databases from inception to January 5, 2023. Two reviewers independently screened search results, extracted relevant data, and evaluated study quality. Statistical analysis was performed using the "metan" command in STATA 16 software. Random-effects or fixed-effects models were chosen based on the magnitude of heterogeneity among studies.

**Results:**

This study included a total of 35 publications, consisting of 23 case–control studies and 12 cohort studies. Meta-analysis of case–control studies showed that alpha-carotene (OR = 0.71, 95% CI: 0.55–0.92), beta-carotene (OR = 0.62, 95% CI: 0.53–0.72), and lutein (OR = 0.82, 95% CI: 0.69–0.97) significantly reduced the risk of gastric cancer, while beta-cryptoxanthin (OR = 0.88, 95% CI: 0.75–1.04) and lycopene (OR = 0.86, 95% CI: 0.73–1.00) showed no significant correlation. Meta-analysis of cohort studies indicated no significant associations between any of the five carotenoids and gastric cancer incidence (alpha-carotene: RR = 0.81, 95% CI: 0.54–1.23; beta-carotene: RR = 0.86, 95% CI: 0.64–1.16; beta-cryptoxanthin: RR = 0.86, 95% CI: 0.64–1.16; lutein: RR = 0.94, 95% CI: 0.69–1.29; lycopene: RR = 0.89, 95% CI: 0.69–1.14).

**Conclusions:**

The relationship between carotenoids and gastric cancer incidence may vary depending on the type of study conducted. Considering that evidence from cohort studies is generally considered stronger than evidence from case–control studies, and high-quality randomized controlled trials show no significant association between carotenoids and gastric cancer incidence, current evidence does not support the supplementation of carotenoids for gastric cancer prevention. Further targeted research is needed to explore the association between the two.

**Supplementary Information:**

The online version contains supplementary material available at 10.1186/s12876-024-03139-5.

## Introduction

Gastric cancer is a heterogeneous and multifactorial disease characterized by high invasiveness. It ranks fifth in terms of incidence and fourth in terms of mortality among all cancers, with over 1 million new cases and nearly 800,000 deaths recorded in 2020 alone. It is a significant public health problem worldwide [1, 2]. Up to 80% of gastric cancer patients exhibit no apparent symptoms in the early stages, and diagnosis often occurs after metastasis has already taken place [3]. Despite significant variations, the 5-year survival rate for gastric cancer remains low and is below 30% in most countries [4, 5]. Therefore, early prevention has become a crucial public health strategy to reduce the incidence and mortality of gastric cancer.

Early prevention of gastric cancer primarily focuses on primary and secondary prevention strategies such as exploring risk and protective factors, improving dietary and lifestyle habits, early screening, and treatment [6]. Among these, the correlation between dietary factors and gastric cancer incidence has been extensively studied. The National Cancer Institute in the United States suggests that the intake of fruits and vegetables can prevent the onset of gastric cancer, while the consumption of grilled, salt-preserved, and smoked foods may contribute to its progression [7]. Most research studies have also indicated that an adequate intake of fruits and vegetables significantly reduces the risk of gastric cancer [8, 9]. Further studies have revealed that carotenoids and other antioxidant substances found in vegetables and fruits play a protective role against gastric cancer [10, 11].

Carotenoids are natural colored pigments widely distributed in nature and are known for their antioxidant and anti-inflammatory properties. There are more than 700 types of carotenoids, including alpha-carotene, beta-carotene, beta-cryptoxanthin, lutein, and lycopene [12, 13]. However, not all carotenoids are capable of reducing the risk of gastric cancer, and there is controversy regarding whether carotenoids can reduce the risk of gastric cancer. Yuan et al., based on the results of a large cohort study involving 18,224 individuals, found a significant correlation between alpha-carotene, beta-carotene, and lycopene levels and a decreased risk of gastric cancer. However, they did not find a significant correlation between serum levels of beta-cryptoxanthin and lutein and the occurrence of gastric cancer [14]. Additionally, Knekt et al. did not find a negative correlation between beta-carotene and gastric cancer incidence in their study [15]. Varis et al. also found that even after supplementing with beta-carotene for five years, the risk of gastric cancer in elderly men did not significantly decrease [16].

In summary, there are conflicting results regarding the relationship between carotenoids and the occurrence of gastric cancer. The role of carotenoids in reducing the risk of gastric cancer remains uncertain. Therefore, this study aims to comprehensively collect relevant research, systematically summarize and analyze the relationship between carotenoids and gastric cancer, in order to provide dietary guidance for future gastric cancer prevention.

## Methods

This systematic review and meta-analysis followed the Preferred Reporting Items for Systematic Reviews and Meta-Analyses (PRISMA) guidelines [17]. The review protocol has been registered with PROSPERO, number CRD42022344012 (https://www.crd.york.ac.uk/prospero/).

### Inclusion and exclusion criteria

#### Patients & diseases

Patients with gastric cancer who were diagnosed by histological examination.

#### Intervention (exposure)

Intake of carotenoids (β-carotene, α-carotene, lycopene, other carotenoids) or blood (plasma or serum) levels of carotenoids. Because the incidence of gastric cancer varies greatly among different age and gender populations, we only included studies that adjusted for age and gender as confounding factors [3, 18]. Other confounding factors were not limited.

#### Outcome

Gastric cancer.

#### Type of study

Cohort studies, case–control studies, and cross-sectional studies.

#### Exclusion criteria

Animal studies, cell research was excluded. Reviews, case reports, opinion articles, conference abstracts, and non‐published data were also excluded.

### Data sources and searches

Systematic searches were conducted in the PubMed, Ovid-Embase, The Cochrane Library, and Web of Science databases, with the search cutoff date set at January 5, 2023. Additionally, the reference lists of included studies were also searched. A combination of free text terms and subject headings was used in the search approach. The search terms and search strategy were as follows: (stomach cancer OR gastric cancer OR gastric carcinoma OR stomach neoplasms OR gastric neoplasms OR stomach carcinoma) AND (lutein OR carotenoids OR carotene OR carotae OR lycopene). Please refer to Additional file 1: Table 1 for the detailed search strategy.

### Data extraction and risk of bias assessment

According to the inclusion and exclusion criteria mentioned above, literature screening and data extraction were performed by two trained researchers. The extracted contents included: 1) Basic information of the included studies: authors, publication year, country, study type, sample size, age, data collection method, gastric cancer diagnostic criteria, data analysis methods, follow-up time, and adjusted confounding factors. 2) Exposure factors: types of carotenoids. 3) Key elements of bias risk assessment.

Based on the Newcastle–Ottawa Scale (NOS), two trained researchers independently assessed the inherent risk of bias in the included studies from three aspects including selection of study population, comparability between groups, and outcome measurement [19]. Scores of 0–3, 4–6, and 7–9 were classified as low, moderate, and high quality, respectively.

### Statistical analysis

Statistical analysis was performed using STATA 16 software. The results were presented as odds ratios (OR) or risk ratios (RR) with corresponding 95% confidence intervals (95% CI). Heterogeneity among the included studies was assessed using the chi-square test with a significance level of α = 0.05, and the magnitude of heterogeneity was determined based on the I^2^ value. A *P*-value > 0.05 indicated that the heterogeneity among the study results was not statistically significant, and a fixed-effect model was used for the meta-analysis. Conversely, if the heterogeneity was found to be statistically significant after further analysis of its sources, a random-effects model was used for the meta-analysis. Additionally, subgroup analyses were conducted based on the types of carotenoids and study design.

## Results

### Literature screening results

**Fig. 1:**
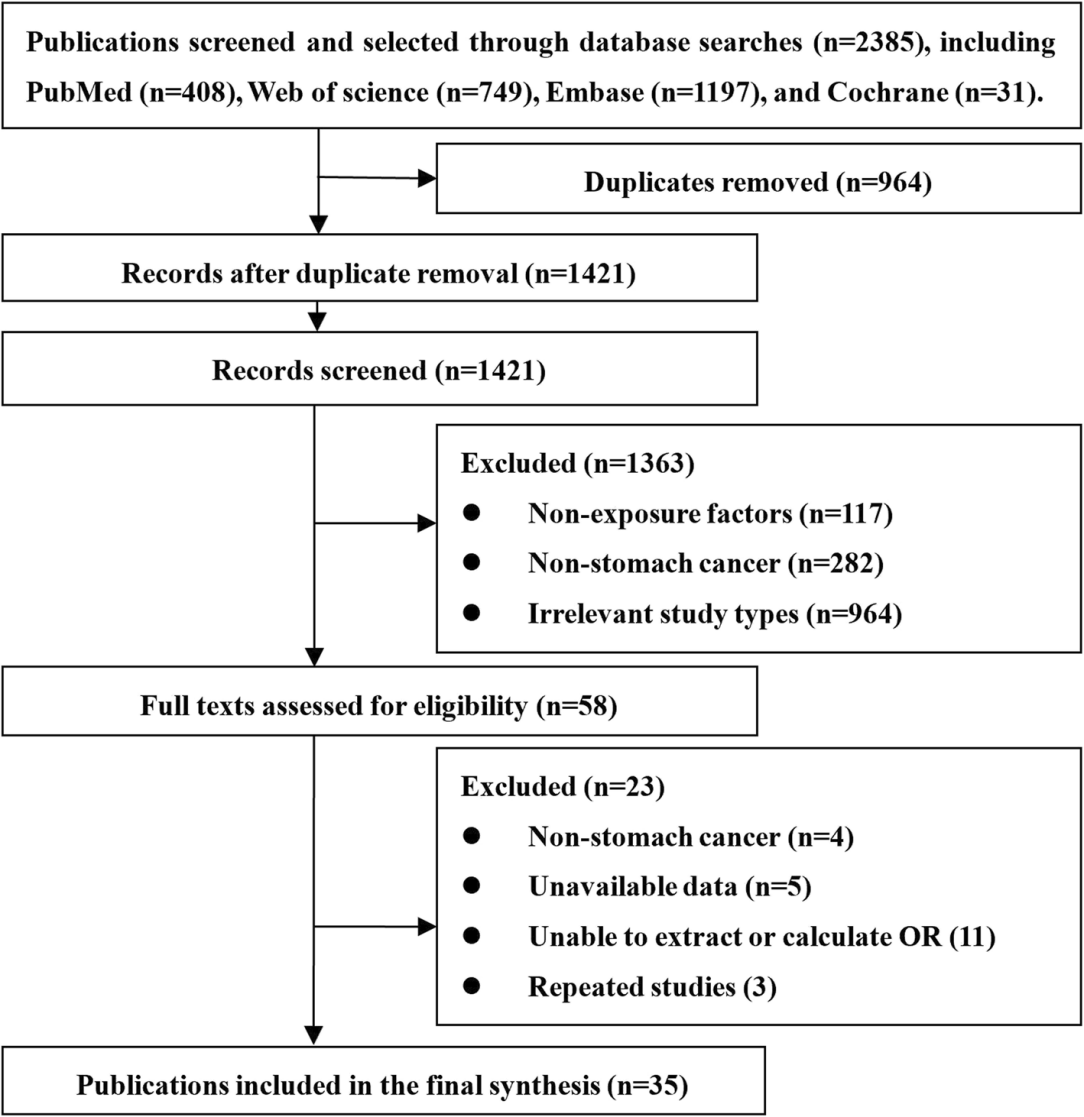
PRISMA Flow chart

A total of 2,385 relevant articles were initially retrieved. After excluding duplicates, articles that did not involve carotenoids as the exposure factor or non-gastric cancer patients (such as precancerous lesions, atrophic gastritis, etc.), and studies that did not meet the appropriate study design (such as reviews, conference abstracts, case reports), 35 studies were ultimately included. The results of the literature screening are summarized in Fig. 1.

### Basic information and risk of bias assessment of included studies

Among the 35 included studies, there were 23 case–control studies [20–42] and 12 cohort studies [14, 16, 43–52]. In the cohort studies, the follow-up time ranged from 5.2 years [51] to 17 years [45], and the sample sizes ranged from 3,123 [43] to 82,002 [48]. The sample sizes of the case–control studies ranged from 223 [25] to 2,747 [29]. The age of the patients ranged from 19 to 89 years, with 9 studies not reporting the age of the patients. All studies used questionnaire surveys to assess exposure factors and gastric cancer was confirmed through histological examination. Multiple logistic regression analysis was conducted in all studies, adjusting for age and gender as confounding factors. The 35 included studies investigated the correlation between five types of carotenoids (alpha-carotene, beta-carotene, beta-cryptoxanthin, lutein, and lycopene) and the risk of gastric cancer. Please refer to Additional file 1: Table 2 for details.

The NOS scores of all 35 studies were ≥ 7, with 19 studies scoring 8 and 17 studies scoring 7, indicating a relatively high quality of included studies. However, the scores for confounding variables and measurement of exposure were relatively low. Please refer to Additional file 1: Table 3 for details.

### Meta-analysis results

#### Alpha-carotene

**Fig. 2:**
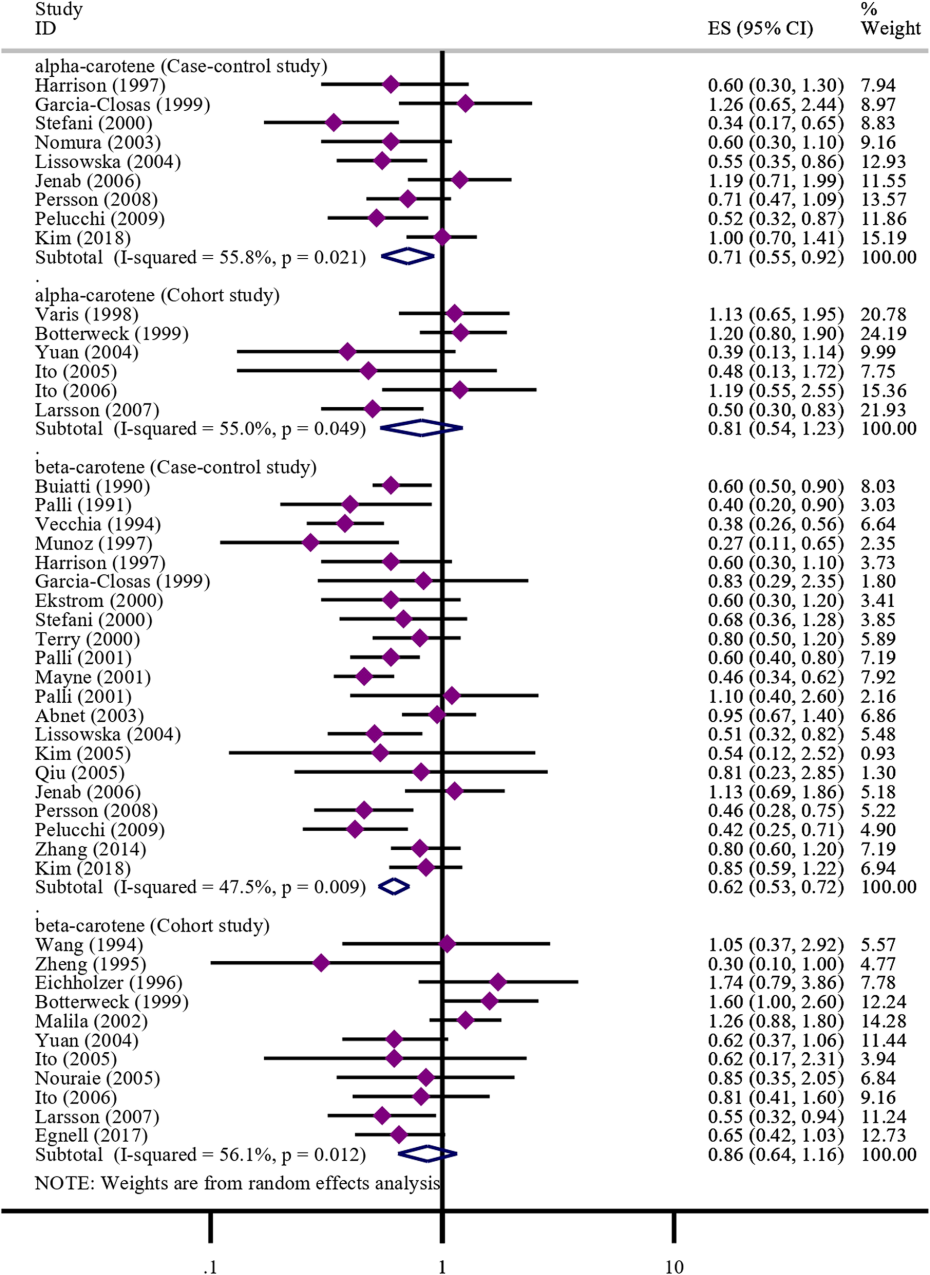
Meta-analysis results of alpha-carotene and beta-carotene

A total of 9 case–control studies and 6 cohort studies reported on the correlation between alpha-carotene and the occurrence of gastric cancer. Due to the statistical heterogeneity test resulting in a *P*-value < 0.05, a random-effects model was used for the meta-analysis. The meta-analysis results based on case–control studies showed that intake of alpha-carotene significantly reduced the risk of gastric cancer (OR = 0.71, 95% CI: 0.55–0.92). However, the meta-analysis results based on cohort studies showed no significant correlation between intake of alpha-carotene and a reduced risk of gastric cancer (RR = 0.81, 95% CI: 0.54–1.23). Please refer to Fig. 2 for further details.

#### Beta-carotene

A total of 21 case–control studies and 11 cohort studies reported on the correlation between beta-carotene and gastric cancer. Due to the statistical heterogeneity test resulting in a *P*-value < 0.05, a random-effects model was used for the meta-analysis. The meta-analysis results based on case–control studies showed that intake of beta-carotene significantly reduced the risk of gastric cancer (OR = 0.62, 95% CI: 0.53–0.72). However, the meta-analysis results based on cohort studies showed no significant correlation between intake of beta-carotene and a decreased risk of gastric cancer (RR = 0.86, 95% CI: 0.64–1.16). Please refer to Fig. 2 for more details.

#### Beta-cryptoxanthin

**Fig. 3:**
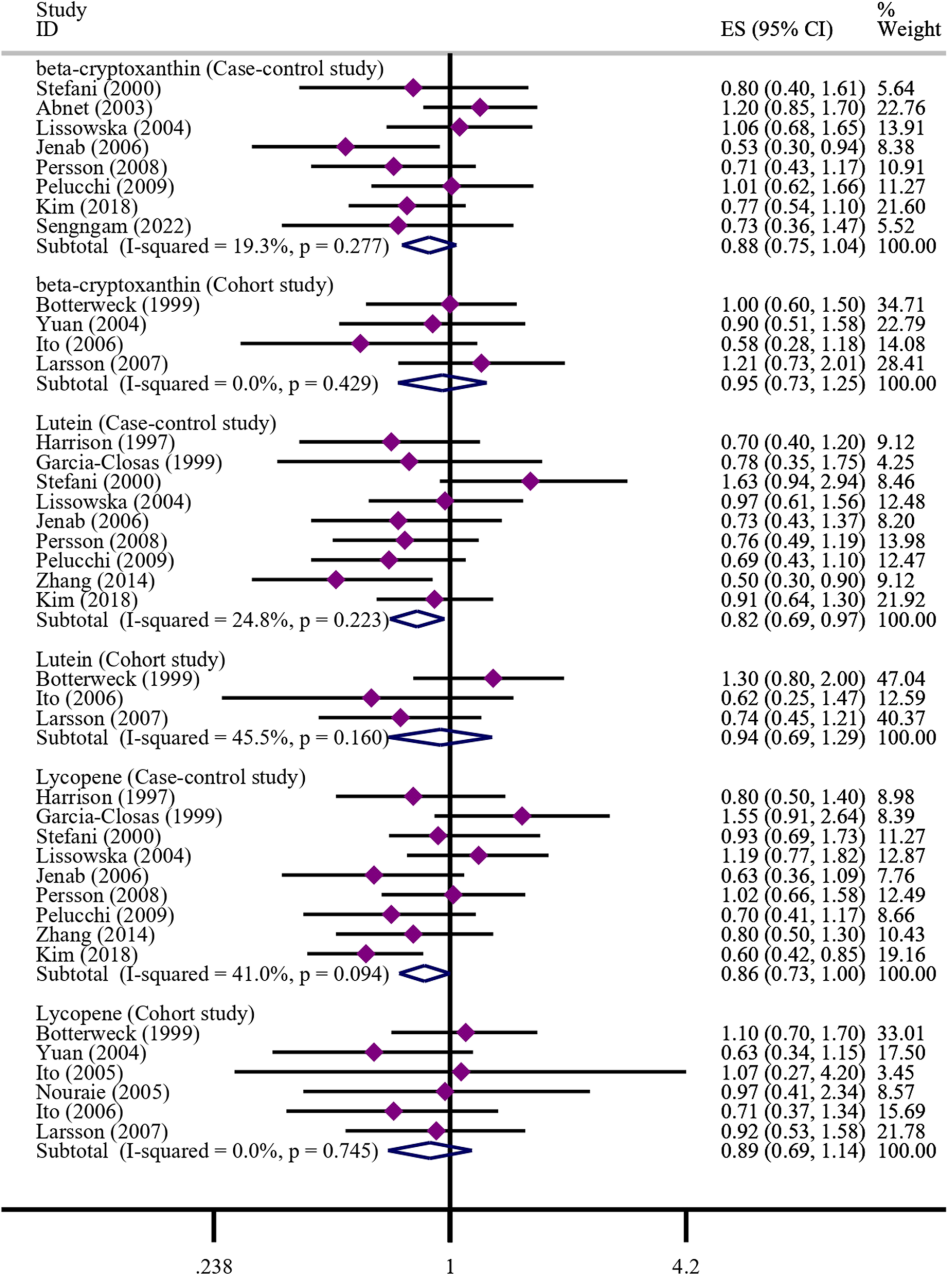
Meta-analysis results of beta-cryptoxanthin, lutein, and lycopene

A total of 8 case–control studies and 4 cohort studies reported on the correlation between beta-cryptoxanthin and gastric cancer. Due to the statistical heterogeneity test resulting in a *P*-value > 0.05, a fixed-effect model was used for the meta-analysis. The meta-analysis results based on both case–control studies and cohort studies showed no significant correlation between intake of beta-cryptoxanthin and a reduced risk of gastric cancer (case–control studies: OR = 0.88, 95% CI: 0.75–1.04; cohort studies: RR = 0.95, 95% CI: 0.73–1.25). Please refer to Fig. 3 for more details.

#### Lutein

A total of 9 case–control studies and 3 cohort studies reported on the correlation between Lutein and the occurrence of gastric cancer. Due to the statistical heterogeneity test resulting in a *P*-value > 0.05, a fixed-effect model was used for the meta-analysis. The meta-analysis results based on case–control studies showed that intake of Lutein significantly reduced the risk of gastric cancer (OR = 0.82, 95% CI: 0.69–0.97). However, the meta-analysis results based on cohort studies showed no significant correlation between intake of Lutein and the occurrence of gastric cancer (RR = 0.94, 95% CI: 0.69–1.29). Please refer to Fig. 3 for more details.

#### Lycopene

A total of 9 case–control studies and 6 cohort studies reported on the correlation between Lycopene and gastric cancer. Due to the statistical heterogeneity test resulting in a *P*-value > 0.05, a fixed-effect model was used for the meta-analysis. The meta-analysis results based on both case–control studies and cohort studies showed no significant correlation between intake of Lycopene and the occurrence of gastric cancer (case–control studies: OR = 0.86, 95% CI: 0.73–1.00; cohort studies: RR = 0.89, 95% CI: 0.69–1.14). Please refer to Fig. 3 for more details.

#### Sensitivity analysis and publication bias of beta-carotene

**Fig. 4:**
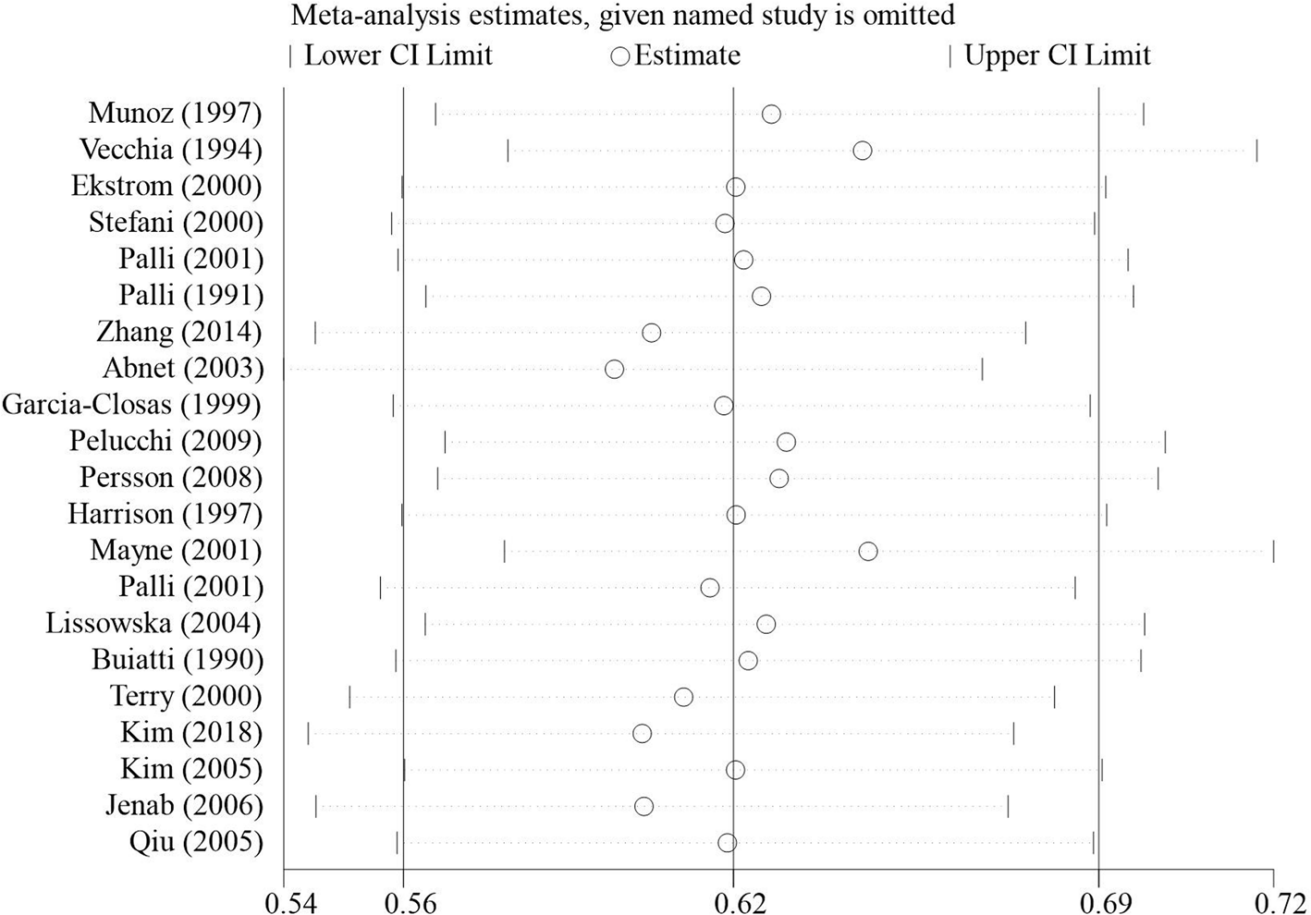
Sensitivity analysis results of beta-carotene

**Fig. 5:**
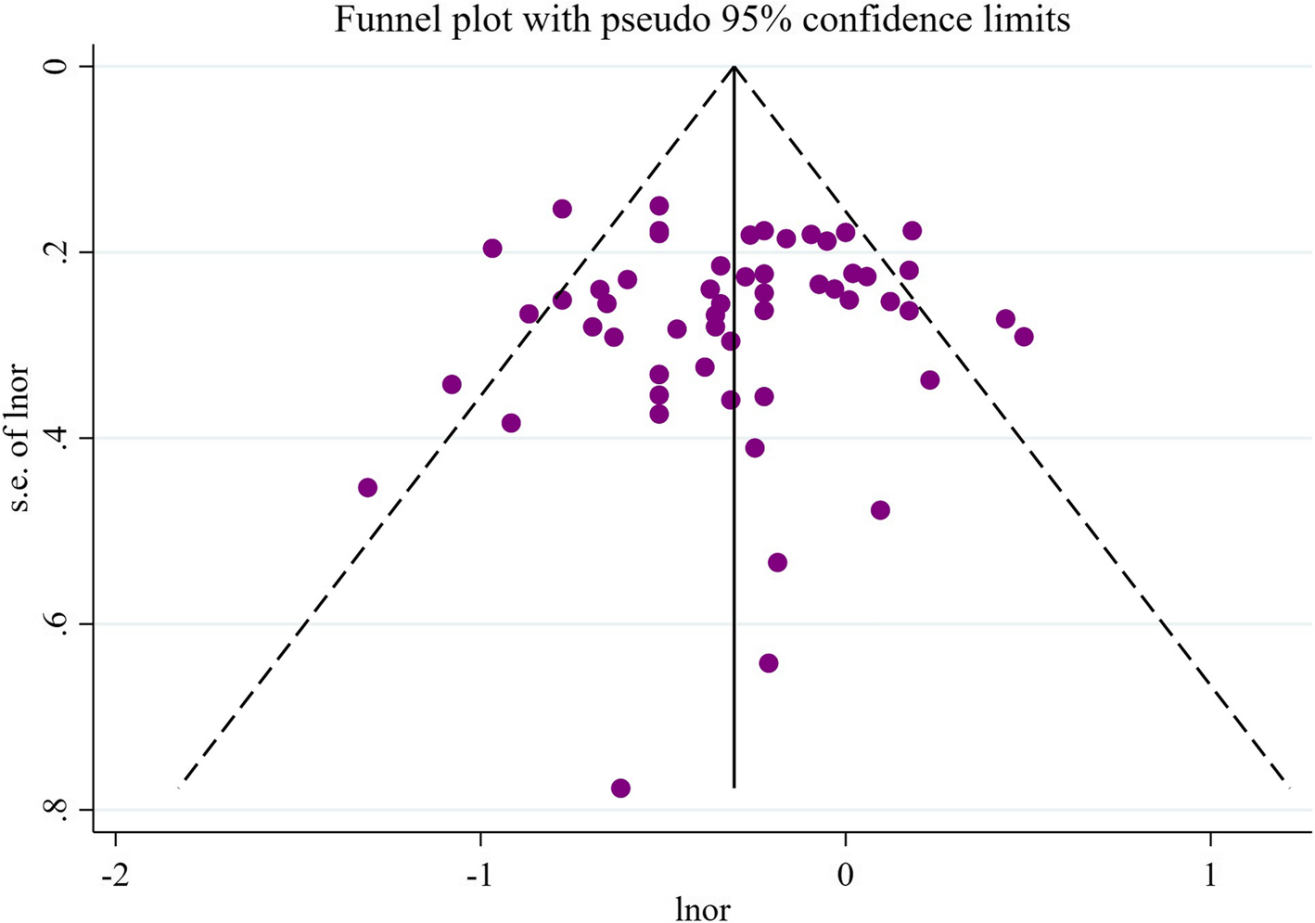
Publication bias results for case–control study

**Fig. 6:**
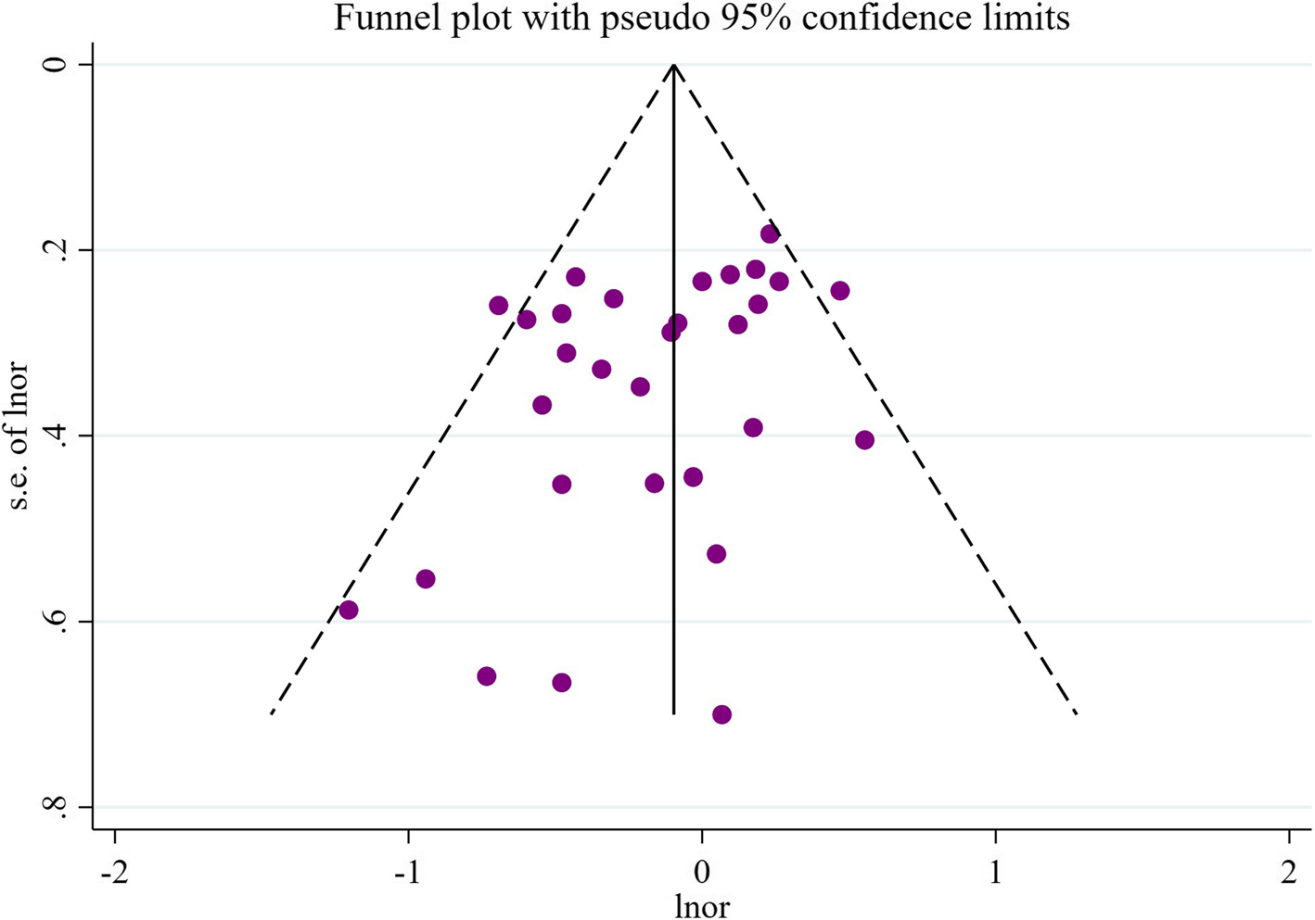
Publication bias results for cohort study

After conducting sensitivity analysis on beta-carotene, we found that the combined values and confidence intervals did not change direction when excluding any specific study, indicating the stability of the meta-analysis results (Fig. 4). Additionally, funnel plots were created based on different study types. The generally symmetrical funnel plots suggest a lower likelihood of publication bias in the current studies (Figs. 5 and 6).

## Discussion

Carotenoids are powerful antioxidants that can interact with reactive oxygen species through oxidation, reduction, hydrogen atom extraction, or addition reactions, reducing oxidative damage to tissue cells [53, 54]. They can also lower the risk of gastric cancer by inhibiting cell proliferation, inducing apoptosis, affecting cell communication, and enhancing immune function [55–58]. Among them, lycopene has been found to inhibit the growth of gastric cancer cells, arrest the cell cycle, induce late-stage apoptosis/necrosis, and decrease mitochondrial membrane potential [59]. Lycopene consists of 89.45% carbon and 10.51% hydrogen, with 11 linear conjugated bonds and 2 non-conjugated double bonds [60]. This unique structure provides strong antioxidant activity, with an effect that is 100 times greater than α-tocopherol and more than twice that of β-carotene [61, 62]. In normal cells, it downregulates inflammation, protects DNA, RNA, and lipids from oxidative damage, and prevents genomic mutations that may lead to cancer [63, 64]. Research has also found that lycopene inhibits the proliferation of Helicobacter pylori-infected gastric adenocarcinoma cells by reducing ROS levels and inhibiting Jak1/Stat3 activation, Wnt/β-catenin signaling, and oncogene expression [64]. Other carotenoids, including α-carotene, β-carotene, lutein, and β-cryptoxanthin, can also influence the development of gastric cancer. For example, β-carotene exhibits anticancer activity by reducing ROS production mediated by NADPH oxidase, activating NF-κB, and regulating the expression of TRAF1 and TRAF2 genes controlled by NF-κB, while inhibiting excessive proliferation of AGS cells [65]. β-cryptoxanthin demonstrates anti-proliferative activity by reducing cell viability, migration, and inducing G0/G1 arrest [66, 67]. Lutein increases the translocation of NADPH oxidase subunit p47Phox to the cell membrane, enhances ROS levels, promotes NADPH oxidase activity, and increases NF-κB activity and apoptotic indices in AGS cells, such as Bax, caspase-3 cleavage, and DNA fragmentation [68]. However, according to the World Cancer Research Fund, not all carotenoids are beneficial for health. Studies have shown that high-dose supplementation of beta-carotene can increase the risk of lung cancer [69, 70]. The effects of carotenoid-containing food supplements on other types of cancer have been considered limited and controversial [71, 72]. In fact, recent research suggests that carotenoids have a dual role in both antioxidant and pro-oxidant activities, making it complex to determine their role in cancer development [71, 73].

Previous case–control studies exploring the association between carotenoids and gastric cancer incidence have indicated that higher intake of carotenoids significantly reduces the risk of gastric cancer even after adjusting for potential confounding factors such as gender, smoking, and Helicobacter pylori infection [28]. However, the results of a randomized, double-blind, placebo-controlled clinical trial involving 22,071 healthy males demonstrated that 12 years of continuous beta-carotene supplementation had no beneficial or harmful effects on reducing the incidence of gastric cancer [74]. These findings are consistent with our research results. Our meta-analysis based on cohort studies revealed no significant correlation between the five types of carotenoids included in the analysis and the occurrence of gastric cancer. In addition, our meta-analysis based on case–control studies showed no significant correlation between beta-cryptoxanthin and lycopene and gastric cancer incidence, while alpha-carotene, beta-carotene, and lutein were negatively associated with gastric cancer incidence. However, it should be noted that except for beta-carotene, the odds ratio values for alpha-carotene and lutein were close to 1, suggesting that their association with gastric cancer occurrence is limited.

Interestingly, when comparing the results of meta-analyses from different study designs, we found that study design appears to have a significant impact on the research outcomes. In contrast to the results of meta-analyses based on case–control studies, the meta-analysis based on cohort studies showed that none of the five carotenoids included in the analysis could reduce the risk of gastric cancer. This is similar to the findings of Friedenreich et al., who found that the correlation between increased intake of vegetables and fruits and reduced cancer risk was only supported by case–control studies, with weaker evidence provided by cohort studies [75]. In fact, prospective cohort studies involve grouping participants based on their exposure characteristics before the occurrence of the outcomes of interest. This minimizes the impact of baseline characteristics, dietary recall, or selection/participation bias. On the other hand, case–control studies are prone to recall bias and selection bias, which can lead to biased results. Therefore, evidence from cohort studies is generally considered stronger than evidence from case–control studies [76–78]. Furthermore, it is challenging to establish a clear protective effect of a specific compound in the development of chronic diseases within a complex diet influenced by many other risk factors. In such cases, conflicting results may also arise [79, 80]. Although the studies we included adjusted for age and gender as confounding factors, there is still a possibility of inadequate control for other confounding factors. Additionally, there are variations in the confounding factors across each study, which may lead to biases in the estimation of risks. Moreover, there are significant differences in the quartiles of carotenoid intake across different studies, which can also affect the calculation of effect sizes. Some studies suggest that inconsistent results may be due to variations in consumption levels of phytochemicals, regional differences, dietary and lifestyle variations, sample size limitations, and data acquisition methods [81–83].

### Strengths and limitations

#### Strengths

1) We only included studies that adjusted for age and gender as confounding factors, minimizing the impact of important confounding factors on the results. 2) We conducted subgroup analyses based on study type and types of carotenoids, resulting in more accurate and representative results. 3) Based on the NOS scoring, we found that the included 35 studies had high quality of evidence, ensuring the reliability of the meta-analysis results. Additionally, we performed publication bias detection and sensitivity analyses to ensure the stability of the meta-analysis results.

#### Limitations

1) Different subtypes of gastric cancer may have varying sensitivities to carotenoids [50]. However, due to the lack of reporting on specific types and stages of gastric cancer in the included studies, we were unable to conduct further subgroup analyses. 2) We only included English-language literature, which may introduce language bias. 3) We did not search for gray literature and conference abstracts, which could potentially introduce publication bias.

## Conclusions

The association between carotenoids and gastric cancer incidence is influenced by the type of study design. Meta-analysis based on case–control studies showed that alpha-carotene, beta-carotene, and lutein can reduce the risk of gastric cancer, while beta-cryptoxanthin and lycopene do not. On the other hand, meta-analysis based on cohort studies showed that there is no significant correlation between different carotenoids and the occurrence of gastric cancer. Considering the evidence from cohort studies is generally stronger than case–control studies, and high-quality randomized controlled trials also show no significant association between carotenoids and gastric cancer, the existing evidence does not support the intake of carotenoids to reduce the risk of gastric cancer. Further high-quality and well-designed experimental studies are needed to investigate the relationship between carotenoids and gastric cancer incidence.

### Supplementary Information

**Additional file 1:** **Table 1. **Literature search strategy. **Table 2. **Basic information of included studies. **Table 3. **Risk of bias assessment results.

## Data Availability

The datasets used and/or analyzed during the current study are available from the corresponding author on reasonable request.

## Abbreviations

PRISMA: Preferred reporting items for systematic reviews and meta-analyses
AOR: Adjusted odds ratio
NOS: Newcastle–ottawa scale
