# Supplementary material for: Not all carotenoids can reduce the risk of gastric cancer: a systematic review with meta-analysis
Source: BMC Gastroenterol. 2024 Jan 29;24:51. doi: 10.1186/s12876-024-03139-5 (PMC10823639; doi:10.1186/s12876-024-03139-5)
Supplement: Supplementary file 1 — Additional file 1: Table 1. Literature search strategy. Table 2. Basic information of included studies. Table 3. Risk of bias assessment results. [file 12876_2024_3139_MOESM1_ESM.docx]

Supplementary Material

**Table 1: Literature search strategy**

| 1.Pubmed  #1: "stomach cancer"[Title/Abstract] OR "gastric cancer"[Title/Abstract] OR "gastric carcinoma"[Title/Abstract] OR "stomach neoplasms"[Title/Abstract] OR "gastric neoplasms"[Title/Abstract] OR "stomach carcinoma"[Title/Abstract] 93,754  #2: "Stomach Neoplasms"[MeSH Major Topic] OR "Stomach Neoplasms"[MeSH Terms] 106,983  #3: #1 OR #2 132,188  #4: "lutein"[Title/Abstract] OR "carotenoids"[Title/Abstract] OR "carotene"[Title/Abstract] OR "carotae"[Title/Abstract] OR "lycopene"[Title/Abstract] 35,363  #5: "Lutein"[MeSH Terms] OR "Carotenoids"[MeSH Terms] OR "zeta Carotene"[MeSH Terms] OR "beta Carotene"[MeSH Terms] OR "Lycopene"[MeSH Terms] 92,131  #6: #5 OR #6 106,806  #7: #3 AND #6 408  2.Web of science  (TS=(stomach cancer OR gastric cancer OR gastric carcinoma OR stomach neoplasms OR gastric neoplasms OR stomach carcinoma)) AND TS=(lutein OR carotenoids OR carotene OR carotae OR lycopene) 749  3.Cochrane  #1: MeSH descriptor: [Stomach Neoplasms] explode all trees 2904  #2: (stomach cancer OR gastric cancer OR gastric carcinoma OR stomach neoplasms OR gastric neoplasms OR stomach carcinoma):ti,ab,kw 10979  #3: #1 or #2  #4: MeSH descriptor: [Luteinization] in all MeSH products 29  #5: MeSH descriptor: [Carotenoids] explode all trees 3858  #6: MeSH descriptor: [Lycopene] explode all trees 273  #7: #4 or #5 or #6 3887  #8: #3 and #7 31  4.Embase  #1: 'stomach cancer':ab,ti OR 'gastric cancer':ab,ti OR 'gastric carcinoma':ab,ti OR 'stomach neoplasms':ab,ti OR 'gastric neoplasms':ab,ti OR 'stomach carcinoma':ab,ti 119051  #2: 'gastric cancer'/exp 142138  #3: 'stomach cancer'/exp 142138  #4: 'gastric carcinoma'/exp 40031  #5: 'stomach neoplasms'/exp 187402  #6: 'stomach carcinoma'/exp 40031  #7: #1 OR #2 OR #3 OR #4 OR #5 OR #6 199794  #8: lutein:ab,ti OR carotenoids:ab,ti OR carotene:ab,ti OR carotae:ab,ti OR lycopene:ab,ti 38877  #9: 'lutein'/exp 8656  #10: 'carotenoids'/exp 177207  #11: 'carotene'/exp 2930  #12: 'lycopene'/exp 7794  #13: #8 OR #9 OR #10 OR #11 OR #12 182924  #14: #7 AND #13 1197 |
| --- |

**Table 2: Basic information of included studies**

| **No.** | **First author** | **Year of publication** | **Place of publication** | **Type of study** | **Sample size** | **Age** | **Information acquisition mode** | **Diagnostic method** | **Statistical method** |
| --- | --- | --- | --- | --- | --- | --- | --- | --- | --- |
| 1 | Munoz | 1997 | Italy | Case–control study | 722/2024 | <75 | Questionnaire method | Histological examination | multiple logistic regression |
| 2 | Zheng | 1995 | USA | Cohort study | 26/34665 | 55-69 | Questionnaire method | Histological examination | multiple logistic regression |
| 3 | Wang | 1994 | China | Cohort study | 16/29584 | 40-69 | Questionnaire method | Histological examination | multiple logistic regression |
| 4 | Vecchia | 1994 | Italy | Case–control study | 723/2024 | 19-74 | Questionnaire method | Histological examination | multiple logistic regression |
| 5 | Ekstrom | 2000 | Sweden | Case–control study | 505/1116 | 40-79 | Questionnaire method | Histological examination | multiple logistic regression |
| 6 | Yuan | 2004 | China | Cohort study | 197/18244 | 45-64 | Questionnaire method | Histological examination | multiple logistic regression |
| 7 | Stefani | 2000 | France | Case–control study | 120/360 | 30-89 | Questionnaire method | Histological examination | multiple logistic regression |
| 8 | Eichholzer | 1996 | Switzerland | Cohort study | 28/3662 | 20-79 | Questionnaire method | Histological examination | multiple logistic regression |
| 9 | Palli | 2001 | Italy | Case–control study | 382/561 | Not reported | Questionnaire method | Histological examination | multiple logistic regression |
| 10 | Palli | 1991 | Italy | Case–control study | 113/1469 | 35-74 | Questionnaire method | Histological examination | multiple logistic regression |
| 11 | Varis | 1998 | USA | Cohort study | 63/29113 | 50-69 | Questionnaire method | Histological examination | multiple logistic regression |
| 12 | Zhang | 2014 | USA | Case–control study | 95/132 | Not reported | Questionnaire method | Histological examination | multiple logistic regression |
| 13 | Malila | 2002 | USA | Cohort study | 126/29113 | 50-69 | Questionnaire method | Histological examination | multiple logistic regression |
| 14 | Abnet | 2003 | USA | Case–control study | 395/1053 | 40-69 | Questionnaire method | Histological examination | multiple logistic regression |
| 15 | Garcia-Closas | 1999 | Spain | Case–control study | 354/354 | 31-88 | Questionnaire method | Histological examination | multiple logistic regression |
| 16 | Nomura | 2003 | USA | Case–control study | 300/446 | 26-95 | Questionnaire method | Histological examination | multiple logistic regression |
| 17 | Larsson | 2007 | Sweden | Cohort study | 139/82002 | Not reported | Questionnaire method | Histological examination | multiple logistic regression |
| 18 | Pelucchi | 2009 | Italy | Case–control study | 230/547 | 22-80 | Questionnaire method | Histological examination | multiple logistic regression |
| 19 | Ito | 2005 | Japan | Cohort study | 20/3182 | 39-79 | Questionnaire method | Histological examination | multiple logistic regression |
| 20 | Ito | 2006 | Japan | Cohort study | 17/3254 | 39-85 | Questionnaire method | Histological examination | multiple logistic regression |
| 21 | Sengngam | 2022 | Viet Nam | Case–control study | 80/146 | >20 | Questionnaire method | Histological examination | multiple logistic regression |
| 22 | Persson | 2008 | Sweden | Case–control study | 511/511 | 25-69 | Questionnaire method | Histological examination | multiple logistic regression |
| 23 | Botterweck | 1999 | Netherlands | Cohort study | 282/3123 | 55-69 | Questionnaire method | Histological examination | multiple logistic regression |
| 24 | Harrison | 1997 | USA | Case–control study | 91/132 | Not reported | Questionnaire method | Histological examination | multiple logistic regression |
| 25 | Mayne | 2001 | USA | Case–control study | 255/687 | 30-79 | Questionnaire method | Histological examination | multiple logistic regression |
| 26 | Palli | 2001 | Italy | Case–control study | 382/561 | Not reported | Questionnaire method | Histological examination | multiple logistic regression |
| 27 | Lissowska | 2004 | Poland | Case–control study | 274/463 | Not reported | Questionnaire method | Histological examination | multiple logistic regression |
| 28 | Buiatti | 1990 | Italy | Case–control study | 1016/1159 | Not reported | Questionnaire method | Histological examination | multiple logistic regression |
| 29 | Terry | 2000 | Sweden | Case–control study | 258/815 | Not reported | Questionnaire method | Histological examination | multiple logistic regression |
| 30 | Kim | 2018 | Korea | Case–control study | 415/830 | Not reported | Questionnaire method | Histological examination | multiple logistic regression |
| 31 | Kim | 2005 | Korea | Case–control study | 136/136 | 57.2 ± 0.84 | Questionnaire method | Histological examination | multiple logistic regression |
| 32 | Egnell | 2017 | France | Cohort study | 9/38812 | 57.1 ± 7.4 | Questionnaire method | Histological examination | multiple logistic regression |
| 33 | Jenab | 2006 | Denmark | Case–control study | 244/645 | 42.8-71.2 | Questionnaire method | Histological examination | multiple logistic regression |
| 34 | Qiu | 2005 | China | Case–control study | 103/133 | 28-85 | Questionnaire method | Histological examination | multiple logistic regression |
| 35 | Nouraie | 2005 | Finland | Cohort study | 243/29133 | 50-69 | Questionnaire method | Histological examination | multiple logistic regression |

**Table 3:** **Risk of bias assessment results**

| **Author** | **Year** | **The selection of participants** | **Confounding variables** | **Measurement of exposure** | **Total score** |
| --- | --- | --- | --- | --- | --- |
| Munoz | 1997 | 4 | 2 | 1 | 7 |
| Zheng | 1995 | 4 | 2 | 1 | 7 |
| Wang | 1994 | 4 | 2 | 1 | 7 |
| Vecchia | 1994 | 4 | 2 | 1 | 7 |
| Ekstrom | 2000 | 4 | 2 | 2 | 8 |
| Yuan | 2004 | 4 | 2 | 2 | 8 |
| Stefani | 2000 | 4 | 2 | 1 | 7 |
| Eichholzer | 1996 | 4 | 2 | 1 | 7 |
| Palli | 2001 | 4 | 2 | 2 | 8 |
| Palli | 1991 | 4 | 2 | 1 | 7 |
| Varis | 1998 | 4 | 2 | 2 | 8 |
| Zhang | 2014 | 4 | 2 | 2 | 8 |
| Malila | 2002 | 4 | 2 | 2 | 8 |
| Abnet | 2003 | 4 | 2 | 2 | 8 |
| Garcia-Closas | 1999 | 4 | 2 | 1 | 7 |
| Nomura | 2003 | 4 | 2 | 2 | 8 |
| Larsson | 2007 | 4 | 2 | 1 | 7 |
| Pelucchi | 2009 | 4 | 2 | 1 | 7 |
| Ito | 2005 | 4 | 2 | 1 | 7 |
| Ito | 2006 | 4 | 2 | 2 | 8 |
| Sengngam | 2022 | 4 | 2 | 1 | 7 |
| Persson | 2008 | 4 | 2 | 2 | 8 |
| Botterweck | 1999 | 4 | 2 | 2 | 8 |
| Harrison | 1997 | 4 | 2 | 2 | 8 |
| Mayne | 2001 | 4 | 2 | 2 | 8 |
| Palli | 2001 | 4 | 2 | 1 | 7 |
| Lissowska | 2004 | 4 | 2 | 2 | 8 |
| Buiatti | 1990 | 4 | 2 | 1 | 7 |
| Terry | 2000 | 4 | 2 | 1 | 7 |
| Kim | 2018 | 4 | 2 | 2 | 8 |
| Kim | 2005 | 4 | 2 | 2 | 8 |
| Egnell | 2017 | 4 | 2 | 2 | 8 |
| Jenab | 2006 | 4 | 2 | 2 | 8 |
| Qiu | 2005 | 4 | 2 | 1 | 7 |
| Nouraie | 2005 | 4 | 2 | 2 | 8 |
